# Cross-validation of an artificial intelligence tool for fracture classification and localization on conventional radiography in Dutch population

**DOI:** 10.1186/s13244-025-02034-1

**Published:** 2025-07-03

**Authors:** Huibert C. Ruitenbeek, Sahil Sahil, Aradhana Kumar, Ravi Kumar Kushawaha, Swetha Tanamala, Saigopal Sathyamurthy, Rohitashva Agrawal, Subhankar Chattoraj, Jasika Paramasamy, Daniel Bos, Roshan Fahimi, Edwin H. G. Oei, Jacob J. Visser

**Affiliations:** 1 Department of Radiology and Nuclear Medicine, Erasmus MC, University Medical Center Rotterdam, Rotterdam, The Netherlands; 2 Qure.ai, Level 7, Oberoi Commerz II, Goregaon East, Mumbai, India; 3 Department of Epidemiology, Erasmus Medical Center Rotterdam, Rotterdam, The Netherlands; 4 Department of Radiology, Massachusetts General Hospital and Harvard Medical School, Boston, MA USA

**Keywords:** Appendicular skeleton, Conventional radiography, Fracture, Artificial intelligence, Validation

## Abstract

**Objectives:**

The aim of this study is to validate the effectiveness of an AI tool trained on Indian data in a Dutch medical center and to assess its ability to classify and localize fractures.

**Methods:**

Conventional radiographs acquired between January 2019 and November 2022 were analyzed using a multitask deep neural network. The tool, trained on Indian data, identified and localized fractures in 17 body parts. The reference standard was based on radiology reports resulting from routine clinical workflow and confirmed by an experienced musculoskeletal radiologist. The analysis included both patient-wise and fracture-wise evaluations, employing binary and Intersection over Union (IoU) metrics to assess fracture detection and localization accuracy.

**Results:**

In total, 14,311 radiographs (median age, 48 years (range 18–98), 7265 male) were analyzed and categorized by body parts; clavicle, shoulder, humerus, elbow, forearm, wrist, hand and finger, pelvis, hip, femur, knee, lower leg, ankle, foot and toe. 4156/14,311 (29%) had fractures. The AI tool demonstrated overall patient-wise sensitivity, specificity, and AUC of 87.1% (95% CI: 86.1–88.1%), 87.1% (95% CI: 86.4–87.7%), and 0.92 (95% CI: 0.91–0.93), respectively. Fracture detection rate was 60% overall, ranging from 7% for rib fractures to 90% for clavicle fractures.

**Conclusions:**

This study validates a fracture detection AI tool on a Western-European dataset, originally trained on Indian data. While classification performance is robust on real clinical data, fracture-wise analysis reveals variability in localization accuracy, underscoring the need for refinement in fracture localization.

**Critical relevance statement:**

AI may provide help by enabling optimal use of limited resources or personnel. This study evaluates an AI tool designed to aid in detecting fractures, possibly reducing reading time or optimization of radiology workflow by prioritizing fracture-positive cases.

**Key Points:**

- Cross-validation on a consecutive Dutch cohort confirms this AI tool’s clinical robustness.
- The tool detected fractures with 87% sensitivity, 87% specificity, and 0.92 AUC.
- AI localizes 60% of fractures, the highest for clavicle (90%) and lowest for ribs (7%).

**Graphical Abstract:**

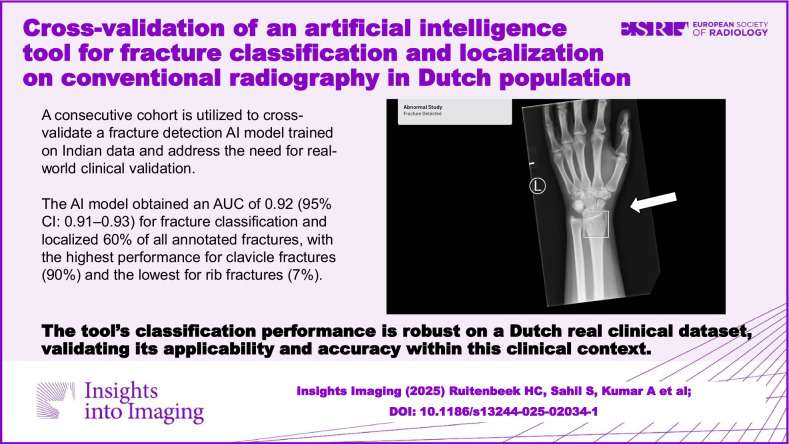

## Introduction

The number of acute fractures is increasing rapidly across the globe [1]. An estimated 178 million fracture cases were reported in 2019 worldwide [2]. Multiple factors contribute to the increased incidence, including road traffic accidents and bone pathologies such as osteoporosis and metastatic cancer.

Conventional radiography is typically the first imaging modality used to assess suspected fractures due to its low cost, minimal radiation and widespread availability. However, radiographs have limitations, including relatively low sensitivity for some fractures and interobserver variability [3]. As a result, 1–3.7% of fractures can be missed on radiographs [4, 5]. The increasing workload and lack of trained radiologists further contribute to wrong diagnosis [6].

Fractures need timely medical attention to reduce the chances of adverse outcomes. Any delay in the identification of fractures can result in complications such as malunion or non-union requiring additional treatment and subsequent costs [7, 8].

Artificial intelligence (AI) applications are expected to play an essential role in healthcare by optimizing limited resources and personnel. Previous studies have shown that AI tools can aid in fracture diagnosis [9, 10], but many were evaluated using stratified and enriched study cohorts, deviating from daily clinical practice [9, 11]. In addition, the training and validation cohorts showed similar demographics, limiting their generalizability. External validation on demographically diverse cohorts is crucial to ensure AI tools perform reliably across different populations and settings. However, few studies have conducted such external validations, leaving a gap in understanding AI performance in diverse clinical environments [12, 13].

Most AI tools have been limited to diagnosing fractures in specific anatomical regions, such as vertebrae, hip or distal radius [14–16]. However, polytrauma cases involve multiple fractures in different locations, highlighting the need for a more comprehensive tool that can detect fractures across various sites. Detection refers to identifying the presence of a fracture in an image, indicated by a score. Localization means pinpointing the fracture’s exact location, shown by a bounding box. In addition to fracture detection, AI can assist radiologists by localizing these fractures, which is particularly challenging in anatomically complex regions where overlapping structures and variable fracture patterns complicate precise identification. Subtle fractures further add to this challenge, as they may be faint, incomplete, or obscured by surrounding anatomy. Previous studies have explored potential benefits and found that using AI tools can reduce reading times [17–21] and improve diagnostic performance [17–19, 22] for radiologists. One pilot study integrating AI into the radiology workflow improved residents’ sensitivity from 84.7 to 91.3% without loss of specificity (97.1% vs. 97.4%) [23].

The AI tool evaluated in this study was developed and trained prior to the start of this study. An important characteristic of the tool under assessment is its development exclusively on radiographs from medical centers in India during its initial development phase, achieving an AUC of 0.9645 for classifying fractures on Indian data [24]. Our external validation aims to assess whether this tool, in its current form, can generalize to radiographs from a Dutch medical center, thereby evaluating its potential applicability as a tool in a different clinical environment. The aim of the study was to externally validate an AI tool trained on a dataset of Indian origin in a Dutch healthcare setting by using a consecutive dataset, focusing on the tool’s ability to classify cases and localize acute fractures.

## Methods

The Institutional Review Board reviewed this study and determined that approval was not required under the Dutch Medical Research Involving Human Subjects Act (WMO) (Study ID: METC-2022-0466) as no intervention or imposed behavior was involved. The obligation to obtain informed consent was waived according to the local protocol.

### Study design and study population

A consecutive dataset of radiographs of patients was collected retrospectively. To ensure balanced representation of common projections, one radiograph per exam was randomly selected, as PACS exams often follow a standard projection sequence (e.g., AP, lateral, oblique). Eligible patients were at least 18 years old and underwent radiography of the appendicular skeleton as the primary indication. Exclusion criteria were incomplete view on the radiograph and absence of the radiologist’s report for the radiograph. Also, we used radiographs that are standard-of-care, excluding radiographs with surgical hardware, calcifications, or bone tumors near the fracture, because the tool was not designed to handle these scenarios during model training. No specific acquisition parameters were required; all included radiographs met local clinical standards, ensuring image quality. Participants were included at Erasmus MC in Rotterdam, the Netherlands. The final consultant radiologist’s report was used for case identification and selection. Data were de-identified before analysis, ensuring compliance with GDPR guidelines. All identifying information (e.g., name, address, date of birth, patient ID) was irreversibly removed, preventing any link to patient identity.

### Specifications of the trained AI tool

The assessed tool is based on a multitask deep neural network that was trained on 1,567,400 radiographs and tested on 200,626 radiographs of patients above 18 years of age. The term “multitask” reflects the network’s ability to perform both classification and localization tasks. Radiographs, collected over 1 year from multiple Indian centers, included clinical radiology reports. Images covered the appendicular skeleton (clavicle, shoulder, humerus, elbow, forearm, wrist, hand, fingers, pelvis, hip, femur, knee, lower leg, ankle, foot, and toes). For the model training, a development reference standard was established by a panel of six radiologists who independently reviewed and annotated the scans while blinded to the clinical radiology reports. Discrepancies between panel annotations and clinical reports were resolved by an adjudicating radiologist, establishing the development reference standard. The model was trained on this dataset, and it was shown to be able to classify and segment fractures in all the body parts of this development set [24].

**Fig. 1:**
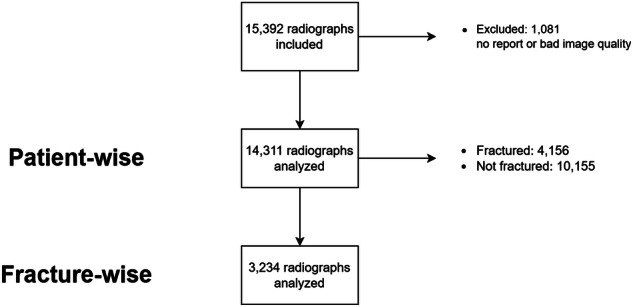
Participant flowchart

The model utilizes a multitask architecture for simultaneous classification and localization. The classification component assigns a probability of fracture presence (0–1), interpreted as positive or negative based on a fixed threshold. For localization, pixel-wise probabilities generate a fracture mask. This enables precise localization of fractures through a segmentation head, which generates pixel-wise data and a fracture mask, which is used to generate a bounding box on the output image. This final output displays both fracture presence and its localization, if detected (Fig. 1).

### Reference standard

In the current external validation study, the reference standard was based on the description of a fracture in the radiology report. These radiology reports were typically produced through a clinical workflow where radiographs were initially reviewed independently by a radiology resident and subsequently supervised and finalized by a senior radiologist with MSK subspecialty training. The reference standard was established by translating the radiologist’s report to bounding boxes, following a protocol to enclose the fracture line tightly from start to end, as accurately as image quality allowed. To ensure accuracy, cases in which the initial readers expressed uncertainty were forwarded for expert review. Additionally, a randomly selected 10% of all annotations underwent independent review by a board-certified musculoskeletal radiologist with more than 10 years of experience. While this reviewing radiologist was not necessarily the same MSK radiologist involved in the clinical workflow, there could be overlaps as the reviewing radiologist is part of the clinical staff. The annotations were performed using a proprietary platform, an online interface with window width adjustment, magnification, and both pen and bounding box tools. Annotators were well-trained and blinded to the AI results. All radiographs were processed within the hospital’s servers. Healed, treated and stress fractures were identified using the radiology reports and marked as positive fractures in the reference standard but not labeled for localization, as they are typically part of follow-up care.

### Analysis

To assess patient-wise metrics, the presence of acute, sub-acute, chronic, treated or healed fractures from the radiology report was expressed binary, regardless of the number of fractures or site.

A fracture-wise analysis was carried out only for acute and sub-acute fractures in cases classified by the AI tool as fractured in the patient-wise analysis. Cases classified as not fractured by the tool do not present bounding boxes to be used in fracture-wise analysis. For each individual fracture, the reference bounding box, which encapsulates the entire fracture as annotated by human annotators, was compared with the AI tool’s output bounding box. This comparison was made based on the Intersection over Union (IoU) metric, which measures the extent of overlap between two bounding boxes. If the IoU value is equal to or greater than 0.10, the reference fracture was considered to be detected adequately by the AI tool, a method previously described by Paramasamy et al [25]. Conversely, if a reference bounding box had an overlap less than 0.10 with any AI tool output bounding box, it was considered undetected by the tool. This comparison allowed for assessing the fracture detection rate, representing the percentage of fracture cases that were successfully localized by the tool, indicating the tool’s ability to accurately localize fractures and detect all present fractures within the radiographic images.

### Statistical methods

This study was intended to include 1000 radiographs per body part (clavicle, shoulder, humerus, elbow, forearm, wrist, hand and finger, pelvis, hip, femur, knee, lower leg, ankle, foot and toe) with approximately 15% fracture cases. With an estimated sensitivity of around 80–90% and specificity of 90–92% a sample size of 15,000 is deemed sufficiently large to evaluate the tool’s sensitivity and specificity overall [17, 19]. This sample size is also likely to provide sufficient statistical power for the secondary anatomical subgroup analysis, at least for the most commonly fractured anatomies.

Sensitivity, specificity, and AUC are the primary metrics, assessed overall and per body part. The tool’s operating point is set at the maximum Youden’s index. Each body part is analyzed independently, treated as a separate study without influencing other diagnostic metrics. As calculations are independent, no multiplicity adjustment is required.

The annotation data and AI tool output were assessed using Python, resulting in the sensitivity, specificity and IoU values. Bootstrapping was used to calculate 95% confidence intervals for these metrics (Python Software Foundation, Python Language Reference, version 3.8. Available at http://www.python.org).

## Results

### Dataset

**Fig. 2:**
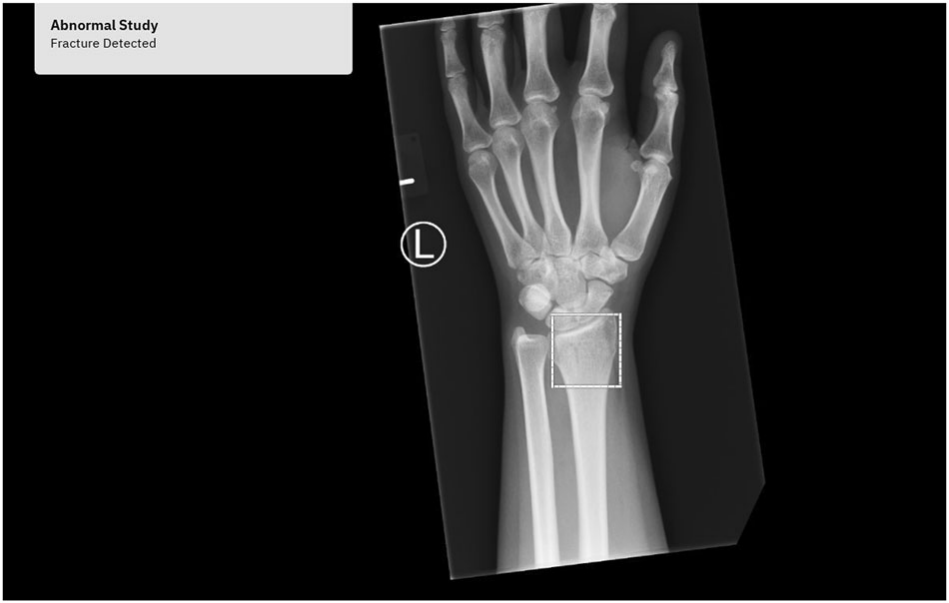
The AI tool outputs a binary classification of fracture presence and localizes fractures at the distal radius using a bounding box

A total of 15,392 radiographs were acquired between January 2019 and November 2022, of which 14,311 were included in the study (Table 1). Median age was 48 years (range 18–98), 7265 were male. 1081 radiographs were excluded based on the absence of reports or not being recognized as supported anatomy by the tool and thus rejected (Fig. 2). The 14,311 radiographs encompassed various abnormalities beyond fractures, including 552 radiographs related to tumors and osteosarcoma, 945 radiographs depicting calcifications, and 3881 radiographs indicating treated and old fractures. Among the total of 14,311 radiographs, 4156 (29%) radiographs presented fractures, while 10,155 (71%) radiographs did not. The expert reader reviewed 1608 cases flagged as uncertain by initial readers and the randomly selected 10%, totaling approximately 21% of radiographs, confirming that all annotations correctly classified fracture presence patient-wise. While some bounding boxes were fine-tuned for accuracy, no significant inconsistencies or errors were identified, and the annotations were deemed reliable for further analysis.

**Table 1:** Baseline characteristics of the study population

	Fracture cases (4156)	Non-fracture cases (10,155)
Age		
< 50 years	2047 (49.3)	5334 (52.5)
≥ 50 years	2109 (50.7)	4821 (47.5)
Sex		
Male	2357 (56.7)	4908 (48.3)
Female	1799 (43.3)	5247 (51.7)

### Patient-wise analysis

The patient-wise outcome measures are summarized in Table 2, which presents sensitivity, specificity and AUC, for the overall dataset as well as for each body part individually, the overall patient-wise sensitivity, specificity and AUC were 87.1% (95% CI: 86.1–88.1%), 87.1% (95% CI: 86.4–87.7%) and 0.92 (95% CI: 0.91–0.93) respectively. The tool showed the lowest performance in the elbow, ribs and hand and fingers exams, while its highest performance was observed for clavicle, femur and hip exams.

**Table 2:** Performance metrics for patient-wise fracture classification, including sensitivity, specificity, and AUC, reported for the overall dataset and each anatomical region

Body part	Sensitivity (95% CI)	Specificity (95% CI)	AUC (95% CI)	Number of fractured cases among the total
Overall	87.1 (86.1–88.1)	87.1 (86.4–87.7)	0.92 (0.91–0.93)	4156/14,311
Femur	93.5 (90.5–95.6)	93.4 (91.1–95.2)	0.96 (0.95–0.98)	355/918
Hip	95.5 (91.6–97.6)	90.3 (87.9–92.4)	0.95 (0.92–0.97)	199/862
Clavicle	89.4 (86.1–91.9)	86.0 (80.4–90.2)	0.94 (0.93–0.96)	433/626
Humerus	89.6 (85.1–92.1)	92.3 (88.7–94.8)	0.94 (0.92–0.96)	241/540
Fibula and tibia	92.2 (89.3–94.3)	89.2 (86.3–91.6)	0.93 (0.92–0.95)	446/984
Wrist	88.1 (84.4–91.0)	86.6 (83.6–89.1)	0.93 (0.91–0.94)	378/975
Ankle	87.5 (84.4–90.1)	87.0 (85.2–88.5)	0.93 (0.91–0.94)	512/2108
Knee	91.2 (86.0–94.6)	90.0 (87.8–91.8)	0.93 (0.90–0.96)	171/1067
Forearm	86.4 (80.8–90.5)	89.3 (85.5–92.3)	0.92 (0.89–0.95)	191/510
Pelvis	86.7 (80.8–91.0)	80.2 (77.4–82.8)	0.91 (0.88–0.94)	173/1008
Shoulder	85.0 (79.5–89.2)	86.8 (84.2–89.0)	0.90 (0.87–0.93)	207/966
Foot and toe	80.8 (74.7–85.8)	80.3 (77.5–82.9)	0.87 (0.84–0.90)	193/1021
Elbow	80.2 (75.6–84.2)	75.2 (72.0–78.2)	0.85 (0.82–0.88)	324/1070
Hands and fingers	78.2 (72.6–83.0)	72.2 (69.5–75.6)	0.84 (0.80–0.86)	239/1059
Ribs	76.6 (67.1–84.0)	70.8 (66.7–74.6)	0.83 (0.78–0.88)	94/597

The table is sorted in descending order of AUC

### Fracture-wise analysis

**Fig. 3:**
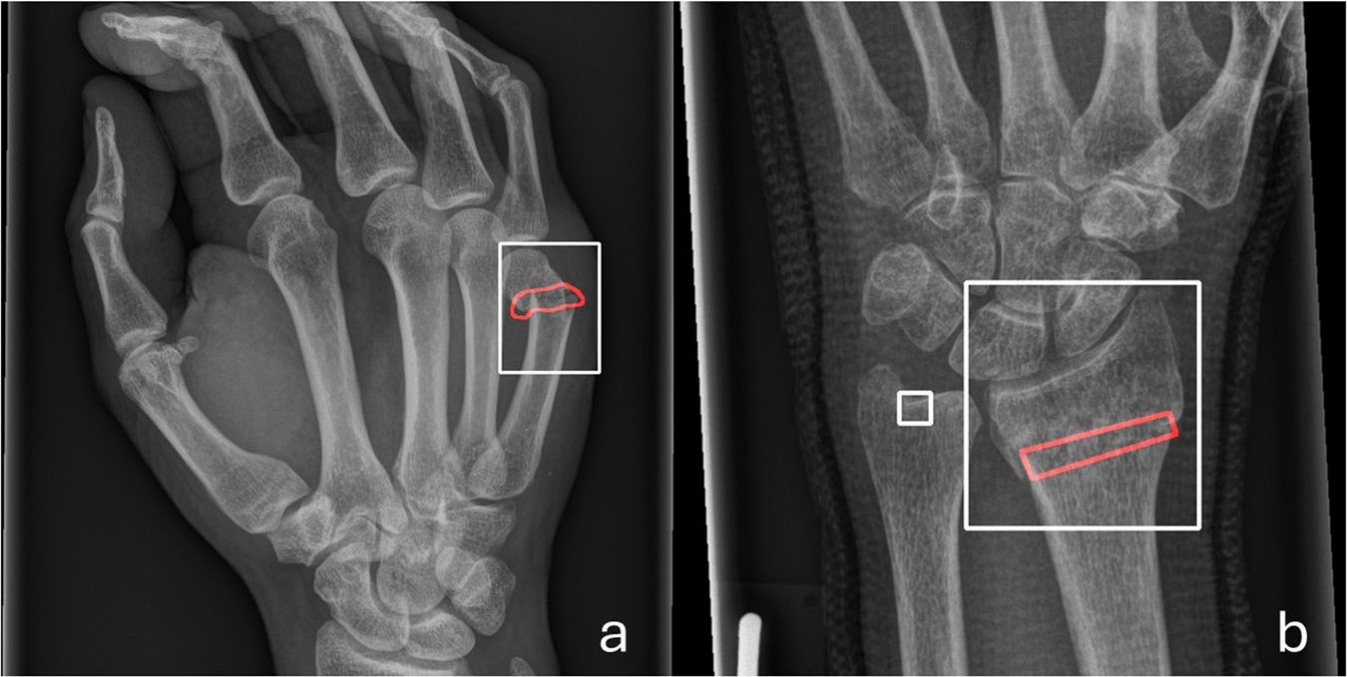
Comparison of reference standard and AI tool fracture localization in two cases. Reference standard annotations are shown in red, and AI tool bounding boxes are shown in white. **a** Fifth metacarpal neck fracture that was annotated in reference standard by a precise annotation. The fracture is localized by the AI tool and fracture-wise analysis results in an IoU of 0.181. **b** Distal radius fracture that was localized with rather wide bounding box. IoU was 0.179. Also, depicted is a false positive model prediction of a fracture mark on the distal ulna

The performance of fracture localization by the tool is displayed in Table 3. The number of 3875 fractures is the sum of all individual bounding box annotations for acute and sub-acute fractures in the reference standard. The mean IoU was calculated for each body part individually and combined. The model demonstrated a higher fracture detection rate in patient-wise analysis but had lower localization performance in fracture-wise analysis. The highest detection rate was observed for clavicle fractures (90%), while the lowest was for ribs (7%). As shown in Fig. 3, some cases with low IoU still sufficiently localize fractures in a clinically meaningful way. These results are presented for the overall dataset and each subgroup individually.

**Table 3:** Fracture-wise analysis results, showing the detection rate for the overall dataset and individual body parts

Body part	Average IoU (95% CI)	Fracture detection rate
Overall	0.25 (0.23–0.27)	60% (2306/3875)
Clavicle	0.30 (0.28–0.31)	90% (367/408)
Femur	0.35 (0.32–0.38)	81% (167/205)
Humerus	0.35 (0.33–0.38)	79% (158/200)
Hip	0.35 (0.31–0.38)	75% (95/126)
Ankle	0.28 (0.27–0.30)	66% (552/838)
Shoulder	0.35 (0.30–0.38)	65% (90/139)
Knee	0.26 (0.22–0.32)	59% (51/86)
Elbow	0.31 (0.28–0.34)	56% (145/261)
Hands and fingers	0.26 (0.23–0.29)	55% (127/231)
Wrist	0.16 (0.14–0.18)	53% (181/339)
Fibula and tibia	0.13 (0.11–0.15)	47% (186/398)
Forearm	0.13 (0.10–0.15)	40% (81/202)
Foot and toe	0.14 (0.12–0.18)	29% (45/155)
Pelvis	0.14 (0.11–0.18)	28% (55/199)
Rib	0.18 (0.16–0.21)	7% (6/88)

A lesion was considered detected when the intersection over union (IoU) between the model’s bounding box and the reference annotation was ≥ 0.10. The table is sorted in descending order of fracture detection rate

*IoU* intersection over Union

## Discussion

Over the past few years, the focus of AI research in radiology has shifted towards studying the implementation and clinical value of AI solutions, with a particular emphasis on their validation on real-world clinical data. This study contributes to the growing body of knowledge regarding the deployment of AI in clinical settings. While external validation of AI tools remains critical, many AI tools still lack such validation. We evaluated the performance of an AI tool trained on Indian data in a Western-European patient population. The tool demonstrated modest performance, with a sensitivity of 87.1%, specificity of 87.1% and an AUC of 0.92. In fracture-wise analysis, the overall detection rate was 60%, with the highest rate observed for clavicle fractures (90%). These results indicate reliable classification performance but highlight the need for further validation to assess generalizability across diverse clinical environments.

Previous literature on fracture detection models has reported varying results. For instance, Cohen et al reported a lower sensitivity of 83% but a higher specificity of 96% for wrist fractures [26]. In comparison, the tool assessed in this study achieved 88.1% sensitivity and a specificity of 86.6% for wrist fractures. However, differences in reference standard may explain part of this variation, as Cohen et al incorporated CT/MRI findings, potentially including fractures that are difficult or impossible to detect on radiographs, while our study relied on routine radiology reports. Bousson et al assessed three AI tools for fracture detection in the appendicular skeleton, with sensitivities ranging from 90.2 to 92.6% and specificities ranging from 70.4 to 92.5% [27]. They also observed variability in AI effectiveness across anatomical regions, attributing lower performance to the anatomical complexity of the region, the diversity of possible fractures, and the small size of the annotations. Similarly, in our study, we observed lower sensitivities for subgroups with smaller structures, particularly in the ribs (76.6%), hand and fingers (78.2%) and foot and toe (80.8%), suggesting that fracture size and annotation challenges may contribute to these differences. Notably, the AUC tool for fracture detection in an Indian dataset was reported to be 0.9645 [24], whereas in our study, it was 0.92 (95% CI: 0.91–0.93). The slightly lower diagnostic performance in our study may be attributed to differences in how the reference standard was defined. Our reference standard incorporated clinical routine, including medical history, which may have led to a more stringent diagnosis compared to previous publications.

While our results are promising, this study has several limitations. The study’s retrospective, single-center design limits its generalizability, and the reference standard was not verified by CT or MRI. As a result, the potential presence of false negative or false positive cases that were correctly annotated by the tool may not have been identified. Additionally, the study excluded cases with bone tumors, calcifications, and those with metal hardware near the fracture site, which are clinically significant. However, such findings are common in clinical practice and may impact AI-assisted fracture detection and the generalizability of the tool. Performance varied across anatomical regions, with lower detection rates and IoU values for fractures in complex areas such as the ribs and pelvis. This may limit the model’s utility in polytrauma cases, where accurate detection across multiple sites is crucial. Another clinically relevant aspect that was not explored here is the impact of AI on reporting times. It remains unclear whether the use of AI decreases or increases radiologists’ reporting times, and whether any efficiency gains persist over time. Future research should investigate this by comparing reading times with and without AI assistance in both controlled reader studies and real-world clinical settings. Furthermore, annotation variability may have influenced localization performance, as even small differences in bounding box placement can significantly impact metrics such as IoU. In addition, although 21% of the annotations were reviewed by an experienced MSK radiologist, including both randomly selected cases and those flagged as uncertain during initial review, the focus on diagnostically challenging cases may have introduced bias. These findings emphasize the need for standardized annotation protocols in AI validation studies to ensure consistency and reliable performance evaluation. Additionally, using only one projection per patient in this study likely underestimated the tool’s performance, as fractures may not always be visible in every projection. Lastly, although we used a 0.10 IoU threshold to assess fracture localization, there are no established clinical guidelines validating a threshold for AI fracture detection tools. This lack of standardization limits interpretability and should be addressed in future studies.

Fracture-wise analysis shows that while the tool performs well in classification at the patient level, localization accuracy is moderate and varies significantly across anatomical regions. This discrepancy is partly due to the small reference annotations compared to larger model-predicted masks and training data limitations. Setting an IoU threshold for evaluation is challenging, as valid detections may be excluded if the predicted bounding box is larger than the reference annotation. Although the fracture detection rate is acceptable, localization needs improvement. More precise annotation during model training, higher-resolution supervision, and techniques such as active learning, uncertainty estimation, and multi-modal embeddings could further refine localization accuracy.

This study provides important insights into the performance of an AI model for fracture detection in a clinical setting. Our external validation of this model highlights its diagnostic accuracy (level 2) and local efficacy (level 7) as defined by Boverhof et al [28]. Moving forward, it will be essential to explore additional levels of efficacy, such as patient outcome efficacy, to assess the model’s impact on patient health and treatment outcomes. Furthermore, the integration of AI models into clinical workflows remains a crucial next step, ensuring that these tools can be seamlessly used by radiologists to support clinical decision-making.

Given its strong classification performance, this tool could act as a triage aid, prioritizing fractured cases and optimizing workflow by archiving non-fractured cases for later review. However, external validation in a Western-European population showed moderate to low localization performance, warranting further research on generalizability and clinical value. Improving localization accuracy could enhance its value for radiology residents and emergency physicians in early fracture detection.

In conclusion, our study demonstrates that the AI tool performs well in patient-wise fracture detection across a clinically representative dataset. However, fracture-wise analysis reveals variability in localization accuracy, particularly in complex anatomical regions, and the exclusion of cases outside standard-of-care may limit generalizability. Future work will focus on improving localization performance, addressing annotation challenges, studying multi-projection cases and cases with atypical bone findings, such as bone tumors and calcifications, and ultimately exploring the integration of AI into clinical workflows.

## Data Availability

The study data were collected retrospectively. The study data are not available.

## Abbreviations

AUC: Area under the curve
AI: Artificial intelligence
IoU: Intersection over Union
